# Performance properties of health-related measurement instruments in whiplash: systematic review protocol

**DOI:** 10.1186/s13643-019-1119-0

**Published:** 2019-08-09

**Authors:** Aidin Abedi, C. A. C. Prinsen, Ishan Shah, Zorica Buser, Jeffrey C. Wang

**Affiliations:** 1 Department of Orthopaedic Surgery, Keck School of Medicine, University of Southern California, 1450 San Pablo St, HC4 - #5400A, Mail Code: 9224, Los Angeles, CA 90033 USA; 2 Department of Epidemiology and Biostatistics, Amsterdam Public Health Research Institute, Amsterdam UMC, location VUmc, Amsterdam, The Netherlands

**Keywords:** Whiplash injuries, Reliability, Validity, Responsiveness, Reproducibility of results, Review, Outcome assessment, Patient-reported outcome measures

## Abstract

**Background:**

Whiplash is a common traumatic cervical injury which is most often a consequence of rear-end motor vehicle accidents. It has been estimated that up to 50% of the whiplash patients suffer from chronic symptoms, resulting in extensive individual and societal burden. Several measurement instruments are used for initial assessment of whiplash and evaluation of response to treatment. However, a comprehensive assessment of the performance of these measures is lacking. Furthermore, there is no consensus on the most relevant outcome domains and their corresponding measurement instruments of choice. This systematic review aims to identify, describe, and critically appraise the performance properties of health-related measurement instruments in whiplash population.

**Methods:**

The following literature databases will be searched from their date of establishment: PubMed, Embase®, MEDLINE, CINAHL Complete, PsycINFO, and HAPI. All original articles evaluating the reliability, validity, responsiveness, and feasibility of health-related measurement instruments in whiplash will be included, without additional restriction on their intended use, source of data, and structure. Risk of bias will be assessed using the COSMIN Risk of Bias checklist. Findings of the studies will be judged against the criteria for good measurement properties, and results from all studies will be qualitatively summarized to generate an overall quality of findings. Overall quality of evidence will be determined using a modified GRADE approach, which will be used in conjunction with the overall quality of results for generation of recommendations. Two reviewers will perform all steps of the review independently. Discrepancies will be discussed between the reviewers, and in case of remaining disagreement, the senior reviewer will make the final decision.

**Discussion:**

This systematic review will summarize the body of literature on health-related measurement instruments in whiplash, aiming to facilitate the selection of high-quality measurement instrument for researchers and physicians. Findings of this study will guide the ongoing efforts for development of a core outcome set.

**Systematic review registration:**

PROSPERO reference number CRD42018070901

**Electronic supplementary material:**

The online version of this article (10.1186/s13643-019-1119-0) contains supplementary material, which is available to authorized users.

## Background

Whiplash is a traumatic neck injury most often associated with rear-end vehicular collisions [1, 2]. This type of accident forces the body to suddenly accelerate forward, causing the neck to hyperextend and then abruptly thrust into flexion [3]. The individual and societal burden of whiplash is extensive and growing. A multi-national review of data on hospital visits between 1970 and 2000 found that the incidence of acute whiplash injuries due to vehicular collisions was approximately 0.3% with an increasing trend [4]. A recent report by the National Highway Traffic Safety Administration estimated that approximately two million rear-end collisions occur every year in the USA, and another study found that 30–40% of these victims experience a neck injury [5, 6]. A report from the Association of British Insurers in 2011 found that over 430,000 whiplash claims are made each year, costing the UK insurers nearly £2 billion per year. Although the resulting injury may resolve acutely, approximately 20–50% of patients report chronic whiplash symptoms, such as neck pain, referred shoulder pain, and paresthesis 1 year following the injury [4, 7, 8].

There is great interest in developing outcome measurement instruments for whiplash injury. These can be used to initially assess the symptoms and severity of injury and to track subsequent changes with treatment [9]. Great strides have been made to explain the pathophysiology of whiplash by seeking objective evidence of physical injury. However, whiplash is currently regarded as a bio-psycho-social phenomenon, with diverse array of outcomes or disease characteristics that need to be measured. To fulfill this growing demand, a wide variety of measurement instruments have been developed. Those are not limited to patient-reported outcomes and cover various pathophysiologic concepts such as cervical mobility, electrophysiology, and imaging. The Quebec Task Force classification of whiplash-associated disorders is one of the most common measures used in the literature [10]. Other whiplash-specific measures have been developed, such as the Whiplash Disability Questionnaire and Whiplash Activity and participation List [11, 12]. Generic measurement instruments, such as visual analog scale for pain, and those developed for non-specific neck pain, such as Neck Disability Index (NDI), are also utilized in this population [13]. While there are many measurement instruments used in assessments, there is no consensus on what outcomes should be measured and which instruments are most optimal to measure these outcomes. Consequently, this lack of standardization leads to difficulty in comparing the results of studies which employ different measures, and particularly limits the application of meta-analysis in systematic reviews [14]. Furthermore, this lack of consensus on outcome measures of choice may lead to selective outcome reporting and bias. Development of core outcome sets is a novel solution to this heterogeneity in outcome measurement [14]. A core outcome set includes the minimum number of outcome measurement instruments that should be used for the evaluation of a specific population [14]. Systematic reviews of outcome measures hold a significant weight in guiding the experts involved in the development of core outcome sets by providing the evidence basis necessary for informed decisions.

### Objectives

The main objectives of this proposed systematic review will be (1) to identify and describe the health-related measurement instruments evaluated for their performance properties (i.e., reliability, validity, and responsiveness) in whiplash; (2) to critically evaluate the methodological quality of studies on measurement properties of those instruments; and (3) to assess the overall quality of health-related measurement instruments in pediatric and adult whiplash populations.

## Methods

### Protocol

Design, conduct, and reporting of this review will be based on the recommendations of the COnsensus-based Standards for the selection of health Measurement INstruments (COSMIN) initiative and the Preferred Reporting Items for Systematic Reviews and Meta-analyses Protocols (PRISMA-P) statement (Additional file 1) [15–17]. The protocol has been registered in the PROSPERO database under reference number 70901.

### Eligibility criteria

All original articles with the main objective of evaluating the reliability, validity, and responsiveness of all outcome measurement instruments in whiplash will be included. Furthermore, studies concerning the development of measurement instruments for/using a whiplash population and those assessing the feasibility aspects of measurement instruments in this population will be eligible. Whiplash is defined according to the Québec Task Force on Whiplash-Associated Disorders as “… an acceleration-deceleration mechanism of energy transfer to the neck. It may result from rear end or side-impact motor vehicle collisions, but can also occur during diving or other mishaps. The impact may result in bony or soft-tissue injuries (whiplash injury), which in turn may lead to a variety of clinical manifestations (Whiplash-Associated Disorders) [10].” Although whiplash falls under the diagnosis of “neck pain-associated disorders,” the latter entity is not the focus of this proposed review [18]. Therefore, studies on patients with neck pain-associated disorders or similar entities will be ineligible, unless at least half of the study population is diagnosed with whiplash. A broad definition of health-related measurement instruments will be used to include not only the outcome measures, but also those concerned with diagnosis, prognosis, and evaluation of disease progress [19]. All health-related measurement instruments will be included, irrespective of their intended use (e.g., diagnostic, evaluative), source of data (e.g., patient-reported, performance-based), and structure (e.g., questionnaire, imaging). Measurement properties are important aspects of the quality of an instrument and include three main domains of reliability, validity, and responsiveness [20]. Each domain includes several measurement properties, which will be defined based on the COSMIN taxonomy and terminology [20]. Domain of reliability addresses “the degree to which the measurement is free from measurement error” and includes internal consistency, measurement error, and reliability itself as a measurement property [20]. Validity is defined as “the degree to which an … instrument measures the construct(s) it purports to measure” and includes content validity, face validity, construct validity, and criterion validity [10, 20]. Responsiveness is “the ability of an … instrument to detect change over time in the construct to be measures [20, 21].” Feasibility is not considered a measurement property. However, it includes practical characteristics of a measure, such as patient or researcher burden, cost, and availability of translations [22]. Details of the eligibility criteria are presented in Table 1.

**Table 1:** Eligibility criteria

Criterion	Explanation
Study designs	Development, adaptation, translation, or evaluation of measurement properties of measurement instruments. Reviews will be initially included for citation tracking.
Population	Patients with clinical manifestation of whiplash defined according to the Québec Task Force on Whiplash-Associated Disorders: “Whiplash is an acceleration-deceleration mechanism of energy transfer to the neck. It may result from rear end or side-impact motor vehicle collisions, but can also occur during diving or other mishaps. The impact may result in bony or soft-tissue injuries (whiplash injury), which in turn may lead to a variety of clinical manifestations (Whiplash-Associated Disorders) [10].” For content/face validity studies: > 50% of the patients with whiplash when patients are involved. For all other studies: > 50% of the whole study population with whiplash OR separate sub-group analysis of patients with whiplash performed.
Severity	Whiplash grade 0–IV according to the Quebec Task Force classification of whiplash-associated disorders [10].
Type of measure	Unrestricted, including but not limited to classifications, patient-reported outcome measures, prediction rules/models, performance-based measures and imaging.
Construct	All health-related constructs addressed in the literature.
Timing	Acute (≤ 3 months) and chronic (> 3 months) phase
Settings	Acute care, rehabilitation, and community
Language	Unrestricted

### Literature sources

The following electronic databases will be searched from their date of implementation: PubMed, Embase®, MEDLINE (via Ovid®), CINAHL Complete (via EBSCOhost®), and PsycINFO (via ProQuest®). Additionally, the Health and Psychosocial Instruments (HAPI) database will be searched via Ovid® for the measurement instruments listing whiplash as a sample or measure descriptor, and source article(s) listed for each instrument will be pooled with the records identified in other databases. References of the identified reviews and original articles that meet the inclusion criteria will be screened for pertinent records that were not captured by the electronic searches.

### Search strategy

The search strategy will be developed by the senior reviewer (AA) who has experience in designing systematic reviews in the fields of clinimetrics and orthopedic surgery. The search will be peer reviewed by other reviewers, experts in the field, and a medical librarian. The search query for HAPI database will include only the keywords related to whiplash: ‘Whiplash*’ OR ‘WAD’. For all other databases, whiplash keywords will be combined with database-specific controlled vocabulary (e.g., MeSH terms for PubMed and EMTREE terms for Embase®) and a validated search filter for finding studies on evaluation of the measurement properties. COSMIN has developed a comprehensive methodological search filter for PubMed, with 97.4% sensitivity and 9.4% specificity in identifying studies on measurement properties [23]. This filter has been translated for Embase® [24], MEDLINE (via Ovid®) [25], and CINAHL [26]. A similar translation will be made for PsycINFO. No limits will be applied regarding the publication type, date, age, or methodology. Although the search will not be limited based on language, articles without an English abstract will not be captured since the search phrases are in English. In order to verify the sensitivity of the search strategy, initial results will be cross-checked against a previous systematic review which utilized a different search strategy [21]. The draft of the search strategy is presented in Additional file 2.

### Data management

Identified records will be pooled and automatically deduplicated using EndNote X7 (Thomson Reuters, Philadelphia, PA). Additional duplicates will be manually identified and compared based on full texts.

### Selection process

A random sample of the records will be screened independently by all reviewers. Kappa statistics will be calculated to assess the inter-interviewer reliability for this sample, and disagreements will be discussed to ascertain whether a uniform set of objective criteria is being applied. The title and abstract of each record will be appraised against the eligibility criteria by at least two independent reviewers (IS and ZB). Full texts of the potentially relevant records will be retrieved and evaluated for eligibility. During the selection process, discrepancies will be discussed between reviewers, and the senior reviewer (AA) will make the final decision in case of remaining disagreement.

### Data extraction

Two independent reviewers (IS and ZB) will perform the data extraction using a predefined online data collection sheet. The senior reviewer (AA) will compare the extracted data between the reviewers, and disagreements will be dealt with in a similar approach planned for the screening process. Data on interpretability and feasibility will be extracted when available: eligibility criteria; patient selection method; patient characteristics (e.g., demographics, pediatric vs. adult, grade of whiplash); characteristics of the observers, experts, and participants of content validity studies; characteristics of the measures (such as method of administration, number of items, and language); settings; countries; response rate; missing items and their method of handling; distribution of scores; proportion of cases with highest and lowest possible scores; minimal important change or difference; hypotheses in validity studies; and results. The content of the outcome measurement instruments covering similar or closely related domains will be analyzed and compared. Relevant World Health Organization’s International Classification of Functioning, Disability and Health (ICF) domain(s) will be assigned to measurement instruments, using the refined ICF linking rules published in 2016 [27]. ICF serves as a framework for uniform description of concepts related to an individual’s health [27]. It includes four main components of “body functions,” “body structures,” “activities and participation,” and “environmental factors” [27]. Each component includes a hierarchy of first- to fourth-level sub-categories [27]. Two independent reviewers (IS and ZB) will judge the domain(s) being covered by each measure, based on the description provided in the article, publication(s) pertaining to the development and elaboration of the measures, published instructions, and the content of the questionnaires. Using this information, first it will be determined if the measure can be linked to an ICF component. Then, relevant ICF components will be selected and first-level ICF categories will be assigned. When possible, more specific ICF categories (second- to fourth-level) will be determined.

### Assessment of risk of bias

The COSMIN Risk of Bias checklist will be used to evaluate the methodological quality of the included studies on measurement properties [28]. This checklist consists of several boxes, each pertaining to a specific measurement property and containing several questions/standards about the design requirements and statistical methods of the studies [28]. For each measurement property in each study, the COSMIN item with the lowest score will indicate the overall methodological quality (i.e., worst-score-counts method) [28, 29]. In agreement with the COSMIN guideline, we will first evaluate the content validity of the included outcome measurement instruments, to be followed by internal structure, if applicable, and the remaining measurement properties [16].

### Assessment of the quality of the outcome measurement instruments and overall quality of evidence

**Fig. 1:**
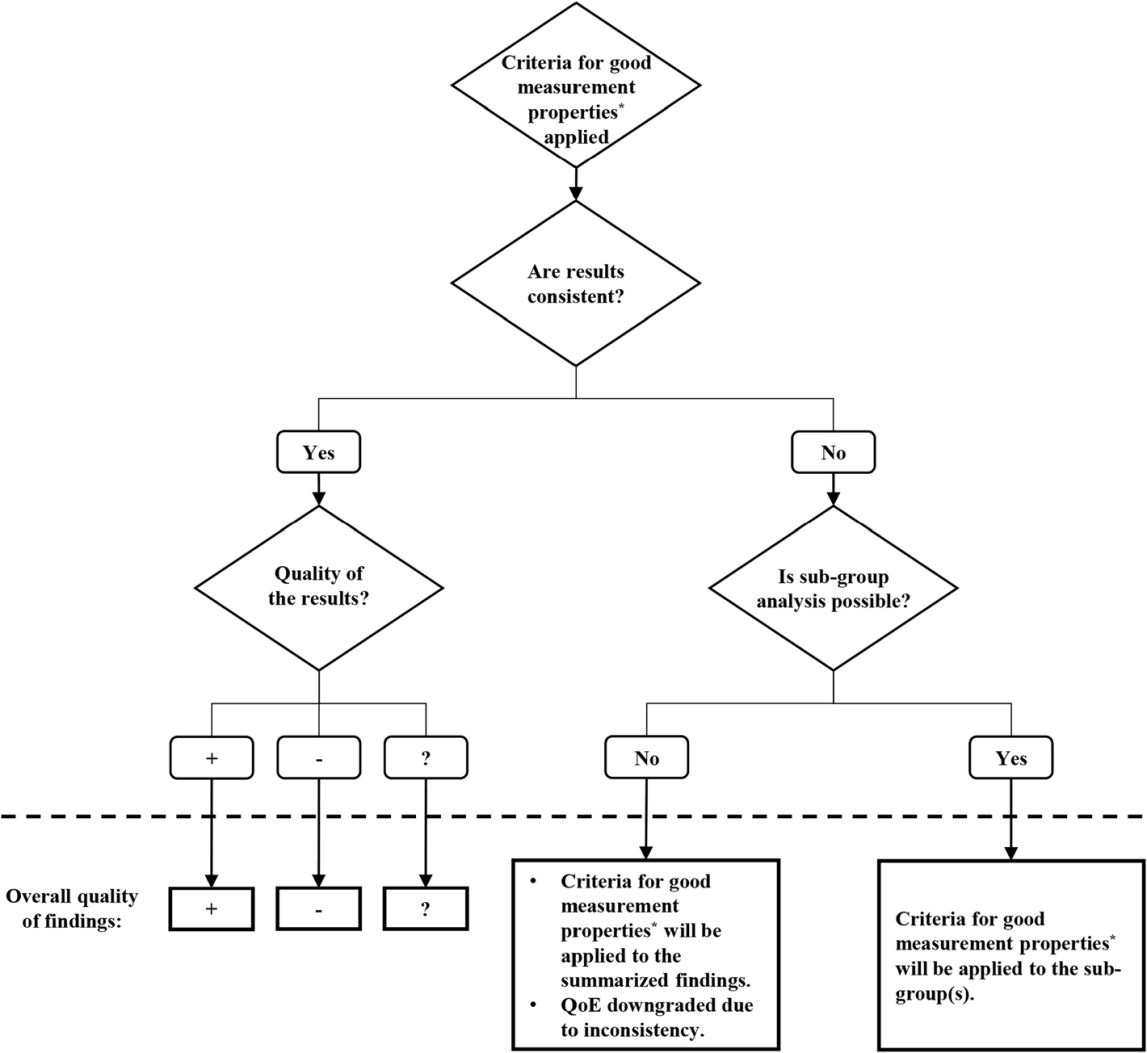
Decision-making algorithm for generation of recommendations. Methodology based on Prinsen et al. [16] and Mokkink et al. [30]. *As described in Table 2

Findings pertaining to the development and feasibility of measurement instruments will be narratively described due to lack of universally accepted quality standards. Prior to quality assessment and synthesis, primary studies on reliability, validity, and responsiveness will be stratified based on methodological approach. Quality assessment will be done in three steps, as follows (Fig. 1):

1. Results of each study will be assessed based on the criteria for good measurement properties by Terwee et al., adapted by Prinsen et al., and rated as sufficient (+), insufficient (−), or indeterminate (?) (Table 2) [14, 16, 31]. For example, if the reliability of NDI score is evaluated in two studies, the ICC values from each study will be rated based on the cut-off point of 0.7 (Table 2). This step will be done separately for each measurement property.
2. The results of all studies will be summarized, to determine whether overall, each measurement property of an instrument is sufficient (+), insufficient (−), inconsistent (±), or indeterminate (?) [16, 30]. This step will be done individually for all measurement properties. When studies are inconsistent, results from pertinent sub-groups of patients/studies will summarized to explain the inconsistency [16, 30]. If not possible, the overall quality will be determined based on majority of the studies, and inconsistency will be accounted for in the next step [16, 30]. In our example scenario, if results of both studies are rated sufficient (+), overall reliability of NDI will be rated sufficient (+) as well.
3. The overall quality of evidence will be rated using the Grading of Recommendations Assessment, Development, and Evaluation (GRADE) approach as modified by Prinsen et al. [16] (Table 3). This approach is explained in detail in COSMIN manual for systematic reviews [30]. In brief, quality of evidence will be downgraded when there is risk of bias (COSMIN Risk of Bias Checklist), inconsistency (if not explained by sub-group analysis), imprecision (based on sample size), and indirectness of evidence [30]. In our example scenario, assuming that both studies had adequate quality (i.e., no risk of bias), their findings were consistent, totals sample size was more than 100, and both studies included only whiplash patients (i.e., direct evidence), quality of evidence for reliability of NDI (determined in step 2) would be high.

**Table 2:** Criteria for evaluation of the quality of results

Measurement property	Rating	Criteria
Structural validity	+	CTT CFA: CFI or TLI or comparable measure > 0.95 OR RMSEA < 0.06 OR SRMR < 0.08^a^
		IRT/Rasch No violation of *unidimensionality*^b^: CFI or TLI or comparable measure > 0.95 OR RMSEA < 0.06 OR SRMR < 0.08 *AND* no violation of *local independence*: residual correlations among the items after controlling for the dominant factor < 0.20 OR Q3’s < 0.37 *AND* no violation of *monotonicity*: adequate looking graphs OR item scalability > 0.30 *AND* adequate *model fit* IRT: χ^2^ > 0.001 Rasch: infit and outfit mean squares ≥ 0.5 and ≤ 1.5 OR Z-standardized values > − 2 and < 2
	?	CTT: not all information for ‘+’ reported IRT/Rasch: model fit not reported
	−	Criteria for ‘+’ not met
Internal consistency	+	At least low evidence^c^ for sufficient structural validity^d^ AND Cronbach’s alpha(s) ≥ 0.70 for each unidimensional scale or subscale^e^
	?	Criteria for “At least low evidence^c^ for sufficient structural validity^d^” not met
	−	At least low evidence^c^ for sufficient structural validity^d^ AND Cronbach’s alpha(s) < 0.70 for each unidimensional scale or subscale^e^
Reliability	+	ICC or weighted Kappa ≥ 0.70
	?	ICC or weighted Kappa not reported
	−	ICC or weighted Kappa < 0.70
Measurement error	+	SDC or LoA < MIC^d^
	?	MIC not defined
	−	SDC or LoA > MIC^d^
Hypotheses testing for construct validity	+	The result is in accordance with the hypothesis^f^
	?	No hypothesis defined (by the review team)
	−	The result is not in accordance with the hypothesis^f^
Cross-cultural validity/measurement invariance	+	No important differences found between group factors (such as age, gender, language) in multiple group factor analysis OR no important DIF for group factors (McFadden’s *R*^2^ < 0.02)
	?	No multiple group factor analysis OR DIF analysis performed
	−	Important differences between group factors OR DIF was found
Criterion validity	+	Correlation with gold standard ≥ 0.70 OR AUC ≥ 0.70
	?	Not all information for ‘+’ reported
	−	Correlation with gold standard < 0.70 OR AUC < 0.70
Responsiveness	+	The result is in accordance with the hypothesis^f^ OR AUC ≥ 0.70
	?	No hypothesis defined (by the review team)
	−	The result is not in accordance with the hypothesis^f^ OR AUC < 0.70

Adapted from Prinsen et al. [16] under a Creative Commons Attribution 4.0 International License (http://creativecommons.org/licenses/by/4.0/). The criteria are updated by Prinsen et al. [16] based on, e.g., Terwee et al. [31] and Prinsen et al. [14]

*AUC* area under the curve, *CFA* confirmatory factor analysis, *CFI* comparative fit index, *CTT* classical test theory, *DIF* differential item functioning, *ICC* intraclass correlation coefficient, *IRT* item response theory, *LoA* limits of agreement, *MIC* minimal important change, *RMSEA* root mean square error of approximation, *SEM* standard error of measurement, *SDC* smallest detectable change, *SRMR* standardized root mean residuals, *TLI* Tucker–Lewis index, *+* sufficient, − insufficient, ? indeterminate

^a^To rate the quality of the summary score, the factor structures should be equal across studies

^b^Unidimensionality refers to a factor analysis per subscale, while structural validity refers to a factor analysis of a (multidimensional) patient-reported outcome measure

^c^As defined by grading the evidence according to the GRADE approach

^d^This evidence may come from different studies

^e^The criteria “Cronbach alpha < 0.95” was deleted, as this is relevant in the development phase of a PROM and not when evaluating an existing PROM

^f^The results of all studies should be taken together, and it should then be decided if 75% of the results are in accordance with the hypotheses

**Table 3:** Modified GRADE approach for evaluation of the overall quality of evidence [16]

Quality of evidence	Definition
High	We are very confident that the true measurement property lies close to that of the estimate of the measurement property
Moderate	We are moderately confident in the measurement property estimate: the true measurement property is likely to be close to the estimate of the measurement property, but there is a possibility that it is substantially different
Low	Our confidence in the measurement property estimate is limited: the true measurement property may be substantially different from the estimate of the measurement property
Very low	We have very little confidence in the measurement property estimate: the true measurement property is likely to be substantially different from the estimate of the measurement property

Reused from Prinsen et al. [14] under a Creative Commons Attribution 4.0 (http://creativecommons.org/licenses/by/4.0/)

### Generation of recommendations

When possible, recommendations will be generated for sub-groups of patients based on age (pediatric versus adult), the severity of whiplash (low-grade versus high-grade), time since injury (acute versus chronic), and other clinically sensible characteristics. Measurement instruments will be categorized according to the COSMIN guideline [16, 30] based on the overall quality of evidence and results, as follows:

Category A: Measures “with evidence for sufficient content validity (any level) AND at least low-quality evidence for sufficient internal consistency” [16]. Measures in this category will be recommended to be used [30].

Category B: Measures “categorized not in A or C” [16]. Further studies on measurement properties of measurement instrument(s) in this category are recommended. If multiple category B measures are available for a construct, the one with the highest evidence for content validity may be used with precaution, until high-quality evidence becomes available [30].

Category C: Measures “with high-quality evidence for an insufficient measurement property” [16]. Use of the measures in this category is not recommended [30]. Modification of these measures may be considered, in order to improve their measurement properties.

### Publication and dissemination

Measurement instruments with a common underlying construct will be grouped and published separately. The target journals will be selected based on the context of each review. Furthermore, the findings of the whole project will be made publicly available through an online platform, as an evidence-based toolkit for selection of measurement instruments for whiplash.

## Discussion

This systematic review will summarize and critically appraise the abounding literature pertaining to the reliability, validity, responsiveness, and feasibility of health-related measurement instruments evaluated in whiplash population. Researchers, physicians, and policy makers in healthcare often need to identify the appropriate measurement instruments for different purposes, such as observation of the natural history of a condition, evaluation of the effectiveness of a treatment, and assessment of the quality of care. This selection process can be complex, and systematic reviews of measurement properties serve as a central component of the evidence-based framework for this purpose [32, 33]. This review will provide evidence-based recommendations on use, optimization, or further evaluation of measures in whiplash. Since the performance of measures may be affected by patient characteristics, recommendations will be generated for sub-groups based on age, severity of whiplash, and other clinically sensible characteristics.

Methodological guidance has been considered in the design of this study, which is expected to increase the quality of evidence derived from this project. This review will utilize three layers of standardized quality assessment, including a robust methodological appraisal checklist. This approach will minimize the subjectivity of the quality assessment process and reduce the risk of bias. The COSMIN checklist was selected for assessment of methodological quality, while alternatives were taken into consideration, such as the Quality Assessment of Diagnostic Accuracy Studies (QUADAS) tool and the Quality Appraisal of Reliability Studies (QAREL) checklist [34, 35]. A pragmatic advantage of COSMIN is that it can be applied to all main categories of measurement properties, while other tools are designed for a specific property, such as reliability or diagnostic accuracy. Although this checklist was designed for patient-reported measures, the standards are generally applicable to other types of measurement instruments [36]. Comprehensiveness of COSMIN prevents the confusion associated with the use of multiple checklists in a review. Our recent content comparison between the COSMIN and QAREL checklists revealed a number of limitations of the latter method: while COSMIN is designed for methodological quality assessment, QAREL contains items related to generalizability, which should be distinguished from methodological quality [37]. Although the developers of QAREL provided guidance for rating the statistical methods, this part of the checklist leaves room for subjectivity [37]. COSMIN checklist is now integrated into a framework for systematic reviews of measurement properties, which includes a modified GRADE approach for overall quality of evidence [16]. The original GRADE criteria are routinely used in systematic reviews of interventional studies. While the original approach is plausible from a methodological standpoint, the modified approach is tailored to address the specifications of clinimetric studies.

In this review, multiple literature databases will be searched to capture the vast majority of the relevant publications. Unlike similar systematic reviews which usually focus on a specific outcome domain, this study will not include any domain-specific keywords in the search. At the cost of increasing the burden of the review, this method will improve the sensitivity of the literature searches and in part the overall quality of the study. Besides, this approach will provide an opportunity to identify the most important methodological flaws in a large sample of clinimetric studies. Meanwhile, measurement instruments available for each domain will be addressed in-depth, by being divided into separate publications, to avoid the over-simplification that is often associated with mega-reviews [19, 28].

There is confusion in the literature regarding the taxonomy and definition of concepts related to measurement. Two key questions should be addressed prior to any measurement: “what to measure” and “how to measure” it [27]. For instance, outcomes are the constructs being evaluated in outcome studies (what to measure), while outcome measures are the tools for this purpose (how to measure) [27]. The focus of this proposed review is indeed on “how to measure,” and “what to measure” needs to be determined on an individual basis at least until a core outcome set is developed for whiplash. Meanwhile, outcome measurement should be distinguished from other purposes of measurement, such as diagnosis, classification, and prognosis [19]. The scope of this review is broad to include not only outcome measures, but also other health-related measurement instruments.

While the importance of having good measurement properties has been emphasized in the literature, those are not the only critical points in the measure selection process, as there are feasibility aspects that should be considered [14, 38]. While this study by itself will serve as a guide for the selection of measures, it is complementary to the ongoing efforts for the development of a core outcome set for whiplash.

## Additional files

Additional file 1: PRISMA-P checklist. (DOCX 36 kb)

Additional file 2: Search strategies. (DOCX 42 kb)

## Data Availability

Not applicable

## Abbreviations

COSMIN: COnsensus-based Standards for the selection of health Measurement Instruments
GRADE: Grading of Recommendations Assessment, Development, and Evaluation
HAPI: Health and Psychosocial Instruments
ICF: International Classification of Functioning, Disability and Health
NDI: Neck Disability Index
PRISMA-P: Preferred Reporting Items for Systematic Reviews and Meta-Analyses Protocols
QAREL: Quality Appraisal of Reliability Studies
QUADAS: Quality Assessment of Diagnostic Accuracy Studies
