# Supplementary material for: Performance properties of health-related measurement instruments in whiplash: systematic review protocol
Source: Syst Rev. 2019 Aug 9;8:199. doi: 10.1186/s13643-019-1119-0 (PMC6688369; doi:10.1186/s13643-019-1119-0)
Supplement: Supplementary file 2 — Search strategies. (DOCX 42 kb) [file 13643_2019_1119_MOESM2_ESM.docx]

**Additional file 2 – Search strategies**

**PubMed search strategy:**

1. Whiplash* OR WAD OR whiplash injuries[MeSH Terms]‎

2. COSMIN search filter for studies on evaluation of the measurement properties [[23](#_ENREF_23)]:

((instrumentation[sh] OR methods[sh] OR Validation Studies[pt] OR Comparative Study[pt] OR “psychometrics”[MeSH] ‎OR psychometr*[tiab] OR clinimetr*[tw] OR clinometr*[tw] OR “outcome assessment (health care)”[MeSH] OR outcome ‎assessment[tiab] OR outcome measure*[tw] OR “observer variation”[MeSH] OR observer variation[tiab] OR “Health ‎Status Indicators”[Mesh] OR “reproducibility of results”[MeSH] OR reproducib*[tiab] OR “discriminant analysis”[MeSH] ‎OR reliab*[tiab] OR unreliab*[tiab] OR valid*[tiab] OR coefficient[tiab] OR homogeneity[tiab] OR homogeneous[tiab] OR ‎‎“internal consistency”[tiab] OR (cronbach*[tiab] AND (alpha[tiab] OR alphas[tiab])) OR (item[tiab] AND (correlation*[tiab] ‎OR selection*[tiab] OR reduction*[tiab])) OR agreement[tiab] OR precision[tiab] OR imprecision[tiab] OR “precise ‎values”[tiab] OR test–retest[tiab] OR (test[tiab] AND retest[tiab]) OR (reliab*[tiab] AND (test[tiab] OR retest[tiab])) OR ‎stability[tiab] OR interrater[tiab] OR inter-rater[tiab] OR intrarater[tiab] OR intra-rater[tiab] OR intertester[tiab] OR inter-‎tester[tiab] OR intratester[tiab] OR intra-tester[tiab] OR interobserver[tiab] OR inter-observer[tiab] OR intraobserver[tiab] ‎OR intra-observer[tiab] OR intertechnician[tiab] OR inter-technician[tiab] OR intratechnician[tiab] OR intra-technician[tiab] ‎OR interexaminer[tiab] OR inter-examiner[tiab] OR intraexaminer[tiab] OR intra-examiner[tiab] OR interassay[tiab] OR ‎inter-assay[tiab] OR intraassay[tiab] OR intra-assay[tiab] OR interindividual[tiab] OR inter-individual[tiab] OR ‎intraindividual[tiab] OR intra-individual[tiab] OR interparticipant[tiab] OR inter-participant[tiab] OR intraparticipant[tiab] ‎OR intra-participant[tiab] OR kappa[tiab] OR kappa’s[tiab] OR kappas[tiab] OR repeatab*[tiab] OR ((replicab*[tiab] OR ‎repeated[tiab]) AND (measure[tiab] OR measures[tiab] OR findings[tiab] OR result[tiab] OR results[tiab] OR test[tiab] OR ‎tests[tiab])) OR generaliza*[tiab] OR generalisa*[tiab] OR concordance[tiab] OR (intraclass[tiab] AND correlation*[tiab]) ‎OR discriminative[tiab] OR “known group”[tiab] OR factor analysis[tiab] OR factor analyses[tiab] OR dimension*[tiab] OR ‎subscale*[tiab] OR (multitrait[tiab] AND scaling[tiab] AND (analysis[tiab] OR analyses[tiab])) OR item discriminant[tiab] ‎OR interscale correlation*[tiab] OR error[tiab] OR errors[tiab] OR “individual variability”[tiab] OR (variability[tiab] AND ‎‎(analysis[tiab] OR values[tiab])) OR (uncertainty[tiab] AND (measurement[tiab] OR measuring[tiab])) OR “standard error of ‎measurement”[tiab] OR sensitiv*[tiab] OR responsive*[tiab] OR ((minimal[tiab] OR minimally[tiab] OR clinical[tiab] OR ‎clinically[tiab]) AND (important[tiab] OR significant[tiab] OR detectable[tiab]) AND (change[tiab] OR difference[tiab])) OR ‎‎(small*[tiab] AND (real[tiab] OR detectable[tiab]) AND (change[tiab] OR difference[tiab])) OR meaningful change[tiab] OR ‎‎“ceiling effect”[tiab] OR “floor effect”[tiab] OR “Item response model”[tiab] OR IRT[tiab] OR Rasch[tiab] OR “Differential ‎item functioning”[tiab] OR DIF[tiab] OR “computer adaptive testing”[tiab] OR “item bank”[tiab] OR “cross-cultural ‎equivalence”[tiab]))‎

3. ‎#1 AND #2‎

**Embase search strategy:**

1. ‎ ‎'whiplash injury'/exp‎ OR whiplash*‎ OR WAD

2. Embase translation of COSMIN search filter for studies on evaluation of the measurement properties [[23](#_ENREF_23), [24](#_ENREF_24)]:

‎'intermethod comparison'/exp OR 'data collection method'/exp ‎OR 'validation study'/exp OR 'feasibility study'/exp OR 'pilot ‎study'/exp OR 'psychometry'/exp OR 'reproducibility'/exp ‎OR reproducib*:ab,ti OR 'audit':ab,ti OR psychometr*:ab,ti ‎OR clinimetr*:ab,ti OR clinometr*:ab,ti OR 'observer variation'/exp ‎OR 'observer variation':ab,ti OR 'discriminant analysis'/exp ‎OR 'validity'/exp OR reliab*:ab,ti OR valid*:ab,ti OR 'coefficient':ab,ti ‎OR 'internal consistency':ab,ti OR (cronbach*:ab,ti AND ('alpha':ab,ti ‎OR 'alphas':ab,ti)) OR 'item correlation':ab,ti OR 'item ‎correlations':ab,ti OR 'item selection':ab,ti OR 'item selections':ab,ti ‎OR 'item reduction':ab,ti OR 'item reductions':ab,ti ‎OR 'agreement':ab,ti OR 'precision':ab,ti OR 'imprecision':ab,ti ‎OR 'precise values':ab,ti OR 'test-retest':ab,ti OR ('test':ab,ti ‎AND 'retest':ab,ti) OR (reliab*:ab,ti AND ('test':ab,ti OR 'retest':ab,ti)) ‎OR 'stability':ab,ti OR 'interrater':ab,ti OR 'inter-rater':ab,ti ‎OR 'intrarater':ab,ti OR 'intra-rater':ab,ti OR 'intertester':ab,ti OR 'inter-‎tester':ab,ti OR 'intratester':ab,ti OR 'interobeserver':ab,ti OR 'inter-‎observer':ab,ti OR 'intraobserver':ab,ti OR 'intertechnician':ab,ti ‎OR 'inter-technician':ab,ti OR 'intratechnician':ab,ti ‎OR 'interexaminer':ab,ti OR 'inter-examiner':ab,ti ‎OR 'intraexaminer':ab,ti OR 'interassay':ab,ti OR 'inter-assay':ab,ti ‎OR 'intraassay':ab,ti OR 'intra-assay':ab,ti OR 'interindividual':ab,ti ‎OR 'inter-individual':ab,ti OR 'intraindividual':ab,ti OR 'intra-‎individual':ab,ti OR 'interparticipant':ab,ti OR 'inter-participant':ab,ti ‎OR 'intraparticipant':ab,ti OR 'kappa':ab,ti OR 'kappas':ab,ti ‎OR 'coefficient of variation':ab,ti OR repeatab*:ab,ti OR ‎‎(replicab*:ab,ti OR 'repeated':ab,ti AND ('measure':ab,ti ‎OR 'measures':ab,ti OR 'findings':ab,ti OR 'result':ab,ti OR 'results':ab,ti ‎OR 'test':ab,ti OR 'tests':ab,ti)) OR generaliza*:ab,ti ‎OR generalisa*:ab,ti OR 'concordance':ab,ti OR ('intraclass':ab,ti ‎AND correlation*:ab,ti) OR 'discriminative':ab,ti OR 'known ‎group':ab,ti OR 'factor analysis':ab,ti OR 'factor analyses':ab,ti ‎OR 'factor structure':ab,ti OR 'factor structures':ab,ti ‎OR 'dimensionality':ab,ti OR subscale*:ab,ti OR 'multitrait scaling ‎analysis':ab,ti OR 'multitrait scaling analyses':ab,ti OR 'item ‎discriminant':ab,ti OR 'interscale correlation':ab,ti OR 'interscale ‎correlations':ab,ti OR ('error':ab,ti OR 'errors':ab,ti AND ‎‎(measure*:ab,ti OR correlat*:ab,ti OR evaluat*:ab,ti ‎OR 'accuracy':ab,ti OR 'accurate':ab,ti OR 'precision':ab,ti ‎OR 'mean':ab,ti)) OR 'individual variability':ab,ti OR 'interval ‎variability':ab,ti OR 'rate variability':ab,ti OR 'variability analysis':ab,ti ‎OR ('uncertainty':ab,ti AND ('measurement':ab,ti ‎OR 'measuring':ab,ti)) OR 'standard error of measurement':ab,ti ‎OR sensitiv*:ab,ti OR responsive*:ab,ti OR ('limit':ab,ti ‎AND 'detection':ab,ti) OR 'minimal detectable concentration':ab,ti ‎OR interpretab*:ab,ti OR (small*:ab,ti AND ('real':ab,ti ‎OR 'detectable':ab,ti) AND ('change':ab,ti OR 'difference':ab,ti)) ‎OR 'meaningful change':ab,ti OR 'minimal important change':ab,ti ‎OR 'minimal important difference':ab,ti OR 'minimally important ‎change':ab,ti OR 'minimally important difference':ab,ti OR 'minimal ‎detectable change':ab,ti OR 'minimal detectable difference':ab,ti ‎OR 'minimally detectable change':ab,ti OR 'minimally detectable ‎difference':ab,ti OR 'minimal real change':ab,ti OR 'minimal real ‎difference':ab,ti OR 'minimally real change':ab,ti OR 'minimally real ‎difference':ab,ti OR 'ceiling effect':ab,ti OR 'floor effect':ab,ti OR 'item ‎response model':ab,ti OR 'irt':ab,ti OR 'rasch':ab,ti OR 'differential item ‎functioning':ab,ti OR 'dif':ab,ti OR 'computer adaptive testing':ab,ti ‎OR 'item bank':ab,ti OR 'cross-cultural equivalence':ab,ti

3. ‎ ‎#1 AND #2

**Medline (via Ovid) search strategy** (using a translation of COSMIN search filter for studies on evaluation of the measurement properties [[23](#_ENREF_23), [25](#_ENREF_25)])**:**

1. (instrumentation or methods).sh.

2. (Validation Studies or Comparative Study).pt.

3. exp Psychometrics/

4. psychometr*.ti,ab.

5. (clinimetr* or clinometr*).tw.

6. exp "Outcome Assessment (Health Care)"/

7. outcome assessment.ti,ab.

8. outcome measure*.tw.

9. exp Observer Variation/

10. observer variation.ti,ab.

11. exp Health Status Indicators/

12. exp "Reproducibility of Results"/

13. reproducib*.ti,ab.

14. exp Discriminant Analysis/

15. (reliab* or unreliab* or valid* or coefficient or homogeneity or homogeneous or "internal consistency").ti,ab.

16. (cronbach* and (alpha or alphas)).ti,ab.

17. (item and (correlation* or selection* or reduction*)).ti,ab.

18. (agreement or precision or imprecision or "precise values" or test-retest).ti,ab.

19. (test and retest).ti,ab.

20. (reliab* and (test or retest)).ti,ab.

21. (stability or interrater or inter-rater or intrarater or intra-rater or intertester or inter-tester or intratester or intra-tester or interobserver or inter-observer or intraobserver or intraobserver or intertechnician or inter-technician or intratechnician or intra-technician or interexaminer or inter-examiner or intraexaminer or intra-examiner or interassay or interassay or intraassay or intra-assay or interindividual or inter-individual or intraindividual or intra-individual or interparticipant or inter-participant or intraparticipant or intra-participant or kappa or kappa's or kappas or repeatab*).ti,ab.

22. ((replicab* or repeated) and (measure or measures or findings or result or results or test or tests)).ti,ab.

23. (generaliza* or generalisa* or concordance).ti,ab.

24. (intraclass and correlation*).ti,ab.

25. (discriminative or "known group" or factor analysis or factor analyses or dimension* or subscale*).ti,ab.

26. (multitrait and scaling and (analysis or analyses)).ti,ab.

27. (item discriminant or interscale correlation* or error or errors or "individual variability").ti,ab.

28. (variability and (analysis or values)).ti,ab.

29. (uncertainty and (measurement or measuring)).ti,ab.

30. ("standard error of measurement" or sensitiv* or responsive*).ti,ab.

31. ((minimal or minimally or clinical or clinically) and (important or significant or detectable) and (change or difference)).ti,ab.

32. (small* and (real or detectable) and (change or difference)).ti,ab.

33. (meaningful change or "ceiling effect" or "floor effect" or "Item response model" or IRT or Rasch or "Differential item functioning" or DIF or "computer adaptive testing" or "item bank" or "cross-cultural equivalence").ti,ab.

34. 1 or 2 or 3 or 4 or 5 or 6 or 7 or 8 or 9 or 10 or 11 or 12 or 13 or 14 or 15 or 16 or 17 or 18 or 19 or 20 or 21 or 22 or 23 or 24 or 25 or 26 or 27 or 28 or 29 or 30 or 31 or 32 or 33

35. exp Whiplash Injuries/

36. Whiplash*.af.

37. WAD.af.

38. 35 or 36 or 37

39. 34 and 38

**CINAHL (Via EBSCO host) search strategy:**

1. (MH “Whiplash Injuries”) OR (TX whiplash*) OR (TX wad)

2. CINAHL translation of COSMIN search filter for studies on evaluation of the measurement properties [[23](#_ENREF_23), [26](#_ENREF_26)]:

TI psychometr* OR TI observer variation OR TI reproducib* OR TI reliab* OR TI unreliab* OR TI valid* OR TI coefficient OR TI homogeneity OR TI homogeneous OR TI “internal consistency” OR AB psychometr* OR AB observer variation OR AB reproducib* OR AB reliab* OR AB unreliab* OR AB valid* OR AB coefficient OR AB homogeneity OR AB homogeneous OR AB “internal consistency” OR (TI cronbach* OR AB cronbach* AND (TI alpha OR AB alpha OR TI alphas OR AB alphas)) OR (TI item OR AB item AND (TI correlation* OR AB correlation* OR TI selection* OR AB selection* OR TI reduction* OR AB reduction*)) OR TI agreement OR TI precision OR TI imprecision OR TI “precise values” OR TI test-retest OR AB agreement OR AB precision OR AB imprecision OR AB “precise values” OR AB test-retest OR (TI test OR AB test AND TI retest OR AB retest) OR (TI reliab* OR AB reliab* AND (TI test OR AB test OR TI retest or AB retest)) OR TI stability OR TI interrater OR TI interrater OR TI intrarater OR TI intra-rater OR TI intertester OR TI inter-tester OR TI intratester OR TI intra-tester OR TI interobserver OR TI inter-observer OR TI intraobserver OR TI intra-observer OR TI intertechnician OR TI inter-technician OR TI intratechnician OR TI intra-technician OR TI interexaminer OR TI inter-examiner OR TI intraexaminer OR TI intra-examiner OR TI interassay OR TI inter-assay OR TI intraassay OR TI intra-assay OR TI interindividual OR TI inter-individual OR TI intraindividual OR TI intra-individual OR TI interparticipant OR TI inter-participant OR TI intraparticipant OR TI intra-participant OR TI kappa OR TI kappa’s OR TI kappas OR TI repeatab* OR AB stability OR AB interrater OR AB inter-rater OR AB intrarater OR AB intra-rater OR AB intertester OR AB inter-tester OR AB intratester OR AB intra-tester OR AB interobserver OR AB inter-observer OR AB intraobserver OR AB intra-observer OR AB intertechnician OR AB inter-technician OR AB intratechnician OR AB intra-technician OR AB interexaminer OR AB inter-examiner OR AB intraexaminer OR AB intra-examiner OR AB interassay OR AB inter-assay OR AB intraassay OR AB intra-assay OR AB interindividual OR AB inter-individual OR AB intraindividual OR AB intra-individual OR AB interparticipant OR AB inter-participant OR AB intraparticipant OR AB intra-participant OR AB kappa OR AB kappa’s OR AB kappas OR AB repeatab* OR ((TI replicab* OR AB replicab* OR TI repeated OR AB repeated) AND (TI measure OR AB measure OR TI measures OR AB measures OR TI findings OR AB findings OR TI result OR AB result OR TI results OR AB results OR TI test OR AB test OR TI tests OR AB tests)) OR TI generaliza* OR TI generalisa* OR TI concordance OR AB generaliza* OR AB generalisa* OR AB concordance OR (TI intraclass OR AB intraclass AND TI correlation* or AB correlation*) OR TI discriminative OR TI “known group” OR TI factor analysis OR TI factor analyses OR TI dimension* OR TI subscale* OR AB discriminative OR AB “known group” OR AB factor analysis OR AB factor analyses OR AB dimension* OR AB subscale* OR (TI multitrait OR AB multitrait AND TI scaling OR AB scaling AND (TI analysis OR AB analysis OR TI analyses OR AB analyses)) OR TI item discriminant OR TI interscale correlation* OR TI error OR TI errors OR TI “individual variability” OR AB item discriminant OR AB interscale correlation* OR AB error OR AB errors OR AB “individual variability” OR (TI variability OR AB variability AND (TI analysis OR AB analysis OR TI values OR AB values)) OR (TI uncertainty OR AB uncertainty AND (TI measurement OR AB measurement OR TI measuring OR AB measuring)) OR TI “standard error of measurement” OR TI sensitiv* OR TI responsive* OR AB “standard error of measurement” OR AB sensitiv* OR AB responsive* OR ((TI minimal OR TI minimally OR TI clinical OR TI clinically OR AB minimal OR AB minimally OR AB clinical OR AB clinically) AND (TI important OR TI significant OR TI detectable OR AB important OR AB significant OR AB detectable) AND (TI change OR AB change OR TI difference OR AB difference)) OR (TI small* OR AB small* AND (TI real OR AB real OR TI detectable OR AB detectable) AND (TI change OR AB change OR TI difference OR AB difference)) OR TI meaningful change OR TI “ceiling effect” OR TI “floor effect” OR TI “Item response model” OR TI IRT OR TI Rasch OR TI “Differential item functioning” OR TI DIF OR TI “computer adaptive testing” OR TI “item bank” OR TI “cross-cultural equivalence” OR TI outcome assessment OR AB meaningful change OR AB “ceiling effect” OR AB “floor effect” OR AB “Item response model” OR AB IRT OR AB Rasch OR AB “Differential item functioning” OR AB DIF OR AB “computer adaptive testing” OR AB “item bank” OR AB “cross-cultural equivalence” OR AB outcome assessment

3. #1 AND #2

**PsycInfo (via ProQuest) search strategy:**

1.‎ Whiplash* OR WAD OR ‎su.Exact("whiplash injuries") OR ‎SU.EXACT.EXPLODE("Whiplash")‎

2. Our translation of COSMIN search filter for studies on evaluation of the measurement properties [[23](#_ENREF_23)]:

cl("Psychometrics & Statistics & ‎Methodology" OR "Research ‎Methods & Experimental Design") ‎OR (psychometr* OR clinimetr* OR ‎clinometr* OR "outcome ‎assessment" OR "outcome ‎measure*‎" OR ‎‎"observer variation" ‎OR reproducib* OR reliab*‎ OR ‎unreliab* OR valid* OR coefficient ‎OR ‎homogeneity OR homogeneous ‎‎OR ‎‎“internal consistency” OR ‎agreement OR precision OR ‎imprecision ‎OR “precise ‎values” ‎OR test-retest OR reliab* OR ‎stability OR interrater OR inter-rater ‎‎OR intrarater OR ‎intra-rater OR ‎intertester OR inter-‎tester OR ‎intratester ‎OR intra-tester OR ‎interobserver OR inter-‎observer OR ‎intraobserver ‎OR intra-‎observer OR ‎intertechnician OR inter-technician ‎OR intratechnician ‎OR intra-‎technician ‎OR interexaminer OR ‎inter-examiner OR intraexaminer ‎OR intra-examiner OR ‎interassay ‎OR ‎inter-assay OR intraassay OR ‎intra-assay OR ‎interindividual OR ‎inter-individual ‎OR ‎intraindividual ‎OR intra-individual OR ‎interparticipant OR inter-participant ‎OR intraparticipant ‎OR ‎intra-‎participant OR ‎kappa OR kappa’s ‎OR kappas OR repeatab*‎ OR ‎generaliza* OR generalisa* OR ‎concordance OR discriminative OR ‎‎“known group” OR “factor analys*” ‎OR dimension* OR ‎subscale* ‎OR “‎item discriminant” ‎OR “interscale ‎correlation*” OR error* OR “‎individual variability” OR “standard ‎error of ‎measurement” OR sensitiv* ‎OR responsive* OR “meaningful ‎change” OR ‎‎“ceiling effect” OR ‎‎“‎floor effect” OR “Item response ‎model” OR IRT OR Rasch OR “‎Differential ‎item functioning” OR ‎DIF OR ‎‎“computer adaptive testing‎” OR “item bank” OR “cross-cultural ‎‎equivalence”‎) OR (‎“cronbach* ‎alpha*” OR “replicab* measure*” ‎OR “replicab* finding*” OR “‎replicab* ‎result*” OR ‎‎“replicab* ‎test*” OR “repeated measure*” OR ‎‎“repeated finding*” OR “repeated ‎result*” OR ‎‎“repeated test*” OR “‎item correlation*” ‎OR “item ‎selection*” OR “item reduction*” ‎OR “Test retest” ‎OR “intraclass ‎correlation*” OR “multitrait scaling ‎analys*” OR “uncertainty measur*” ‎OR “variability ‎analys*” OR “‎variability value*” OR “minimal* ‎important change” OR “minimal* ‎important difference” ‎OR “minimal* ‎significant change” OR “minimal* ‎significant difference” OR “minimal* ‎detectable ‎change” OR “minimal* ‎detectable difference” OR “clinical* ‎important change” OR “clinical* ‎important ‎difference” OR “clinical* ‎significant change” OR “clinical* ‎significant difference” OR “clinical* ‎detectable ‎change” OR “clinical* ‎detectable difference” OR “small* ‎real change” OR “small* real ‎difference” OR ‎‎“small* detectable ‎change” OR “small* detectable ‎difference”‎) OR ‎‎(SU.EXACT.EXPLODE("Measure‎ment") OR ‎SU.EXACT.EXPLODE("Error ‎Analysis") OR ‎SU.EXACT.EXPLODE("Test ‎Construction") OR ‎SU.EXACT.EXPLODE("Interrater ‎Reliability") OR ‎SU.EXACT.EXPLODE("Content ‎Analysis") OR ‎SU.EXACT.EXPLODE("Error of ‎Measurement") OR ‎SU.EXACT.EXPLODE("Factor ‎Structure") OR ‎SU.EXACT.EXPLODE("Testing ‎Methods") OR ‎SU.EXACT.EXPLODE("Statistical ‎Reliability") OR ‎SU.EXACT.EXPLODE("Consistency (Measurement)") OR ‎SU.EXACT.EXPLODE("Computer ‎Assisted Testing") OR ‎SU.EXACT.EXPLODE("Factor ‎Analysis") OR ‎SU.EXACT.EXPLODE("Prediction"‎‎) OR ‎SU.EXACT.EXPLODE("Statistical ‎Validity") OR ‎SU.EXACT.EXPLODE("Prediction ‎Errors"))‎

3. #1 AND #2
